# Demystifying the gender of patient after allogeneic bone marrow transplantation from an opposite-sex donor: A case report

**DOI:** 10.1097/MD.0000000000043146

**Published:** 2025-07-04

**Authors:** Jie Teng, Xiaojuan Liu, Li Chang, Guanglu Che, Qiuxia Yang, Shuyu Lai, Jiaxin Duan, Hui Jian, Fang Liu

**Affiliations:** a Department of Laboratory Medicine, West China Second University Hospital, and Key Laboratory of Obstetric and Gynecologic and Pediatric Diseases and Birth Defects of Ministry of Education, Sichuan University, Chengdu, China.

**Keywords:** azoospermia, case report, heterosexual bone marrow transplantation, sexual dysfunction, Y-chromosome microdeletion

## Abstract

**Rationale:**

Bone marrow transplantation (BMT) is an established therapy for hematological malignancies. In recent years, with the advancement of medical research, evaluating the efficacy of BMT in recipients by detecting the presence or absence of chromosomes from an opposite-sex donor has been widely recognized. Numerous cases have shown that the sex hormone levels of recipients who receive BMT from the opposite sex will change significantly. However, whether such hormonal changes will impair sexual function or lead to physiological sex-related manifestations, or even cause changes in the biological sex of BMT recipients, these questions make people cannot help but think deeply. Herein, we report a case of a male patient who received allogeneic BMT from a female donor, demonstrating that transplantation only replaced the recipient’s hematopoietic stem cells and did not change the patient’s biological sex.

**Patient concerns:**

We report a 22-year-old male who was diagnosed with azoospermia during a recent physical examination and sought medical attention at our hospital.

**Diagnoses:**

Semen examination showed no sperm, and the analysis of the peripheral blood chromosome karyotype is 46,XX. The preliminary clinical diagnosis is azoospermia with sexual dysfunction.

**Interventions:**

The results of Y-chromosome microdeletion in peripheral blood could not be interpreted. After reviewing the patient’s history, we only found that he had received a BMT from an opposite-sex donor for chronic myelocytic leukemia 14 years ago, when he was only 8 years old. The peripheral blood and buccal mucosal swabs were collected for Y-chromosome microdeletion examination again. At this point, the truth has finally come to light: At this point, the etiology became clear: The patient’s peripheral blood showed chimeric DNA from both the BMT donor and recipient, while the buccal mucosal swabs—originating from embryonic development—accurately reflected his pretransplantation genetic profile.

**Outcomes:**

The patient, being only 22 years old and having no intention of getting pregnant at present, received no further medical treatment.

**Lessons:**

This case report demonstrates that while sex-mismatched BMT may alter secondary sexual characteristics, it does not fundamentally change the recipient’s genetic sex or core gender identity.

## 1. Introduction

Chronic myelogenous leukemia (CML) is a malignant neoplasm resulting from clonal proliferation of hematopoietic stem cells in the bone marrow, accounting for approximately 15% of all leukemia cases and representing one of the 3 major types of leukemia in China.^[1,2]^ CML is typically characterized by abnormal elevation of peripheral white blood cells, particularly neutrophils and mature granulocytes, as well as increased levels of eosinophils and basophils. A significant hallmark of CML is the presence of a specific genetic abnormality in nearly all cases of the disease. This abnormality is the BCR-ABL1 gene rearrangement, which involves a translocation between chromosomes 9 and 22. This translocation results in the fusion of the BCR (breakpoint cluster region) gene on chromosome 22 with the ABL1 (Abl proto-oncogene 1, non-receptor tyrosine kinase) gene on chromosome 9.^[3,4]^ The fusion of these two genes leads to the production of a hybrid BCR-ABL1 protein, which has constitutive tyrosine kinase activity. This unregulated activity is believed to be a major driver of the uncontrolled proliferation of myeloid cells seen in CML.

Bone marrow transplantation (BMT) is an intravenous infusion of hematopoietic stem and progenitor cells to rebuild the hematopoietic function and immune system of patients, and has become an effective method to treat leukemia and other hematologic malignant diseases.^[5]^ With the deepening of research related to BMT, China’s expert consensus on allogeneic hematopoietic stem cell transplantation for hematologic diseases explicitly states that after successful human leukocyte antigen matching, sex-mismatched transplants may offer distinct immunological advantages over sex-matched transplants .^[6]^

Sex-mismatched BMT involves transferring hematopoietic stem cells from a donor to a recipient of the opposite sex. Beyond the benefits of sex-matched transplants, its most significant advantage is enabling unique engraftment monitoring.^[7]^ By detecting donor-derived sex chromosomes in the recipient’s bloodstream, physicians can effectively evaluate the success of the transplant. This approach provides a valuable tool for long-term surveillance, offering real-time insights into donor cell engraftment and persistence. However, this procedure may significantly impact both recipients’ and donors’ sexual health and gender-related physiology due to complex interactions between hormonal shifts, immune reconstitution, and systemic physiological changes post-transplantation.^[8]^

Infertility is a global health problem that affects millions of people of reproductive age. Available data indicate that 48 million couples and 186 million people worldwide are living with infertility.^[9]^ The etiology of male infertility is multifaceted, encompassing genetic factors, abnormal reproductive organ development, psychological stress, lifestyle habits, etc. Specifically, 10% to 15% of cases are attributed to sperm defects stemming from genetic abnormalities. Chromosome karyotype analysis^[10,11]^ and Y-chromosome microdeletion^[12]^ in peripheral blood are important methods to assist in the diagnosis of hereditary infertility, which can provide reference for clinicians to choose different assisted reproductive technologies and avoid genetic defects inherited from their parents.^[13]^ However, for patients with special medical history, such as BMT, organ transplantation, allogeneic blood transfusion, etc, the detection results of peripheral blood genes may have the possibility of misdiagnosis.

In this study, we presented a case of azoospermia following sex-mismatched BMT. Semen analysis and sex hormone testing revealed abnormalities,while karyotyping showed a 46,XX result. The preliminary clinical diagnosis was azoospermia. Y-chromosome microdeletion in peripheral blood was detected by fluorescence polymerase chain reaction, and it was found that multiple fluorescence channels had curve warping, the cycle threshold value was within the critical range of 34 to 36, which could not accurately interpret the negative or positive. However, the buccal mucosal swabs showed the SRY gene was presence, and no missing site was detected. The results indicate that after BMT, the patient’s peripheral blood contains genetic information from female donor, and at this time, his gender and reproductive function cannot be evaluated only based on the results of peripheral blood testing. In this case, the somatic cells can more accurately reflect the patient’ s genetic information before transplantation because it originates during the embryonic. We hope this case highlights the importance of cautious interpretation of Y chromosome testing in azoospermic patients with a history of sex-mismatched BMT.

## 2. Case presentation

A male patient was diagnosed with CML at the age of 7 at a local hospital. Leukemia fusion gene testing in bone marrow samples showed that BCR ABL p210 was positive. After several months of treatment with imatinib, a molecularly targeted drug, the patient underwent radiotherapy and chemotherapy pretreatment followed by BMT one year after diagnosis due to successful bone marrow matching with a volunteer from the China Bone Marrow Bank. After transplantation, he took immunosuppressants regularly for two years. He has been off the drug for more than 14 years, and up to now, there have been no signs of CML relapse. Furthermore, at age 13, he developed hypothyroidism requiring long-term levothyroxine (Euthyrox) to replace therapy. Two months ago, he sought treatment at our hospital after azoospermia was detected during a physical examination.

**Figure 1.**
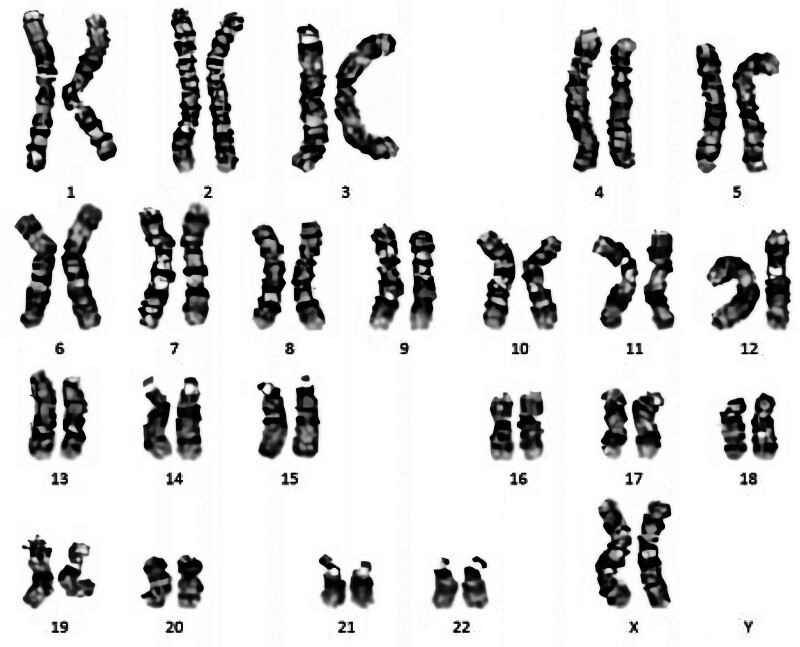
The result of chromosome karyotype analysis in peripheral blood.

Scrotal color Doppler ultrasonography revealed bilateral testicular atrophy and small seminal vesicles. The biochemical results of seminal plasma showed that the total citric acid was lower than normal (as shown in Table 1), and the routine semen analysis in our hospital showed that the total number of sperm and sperm concentration were both 0 (as shown in Table 2). The levels of sex hormone examination indicated that (follicle stimulating hormone: 13.7 mIU/mL, reference range < 10 mIU/mL) was higher than normal male. The results of chromosome G band analysis were 46,XX (as shown in Fig. 1).

**Table 1:**
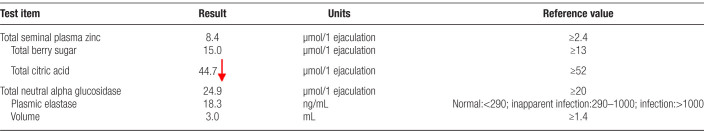
Biochemical results of seminal plasma.

**Table 2:**
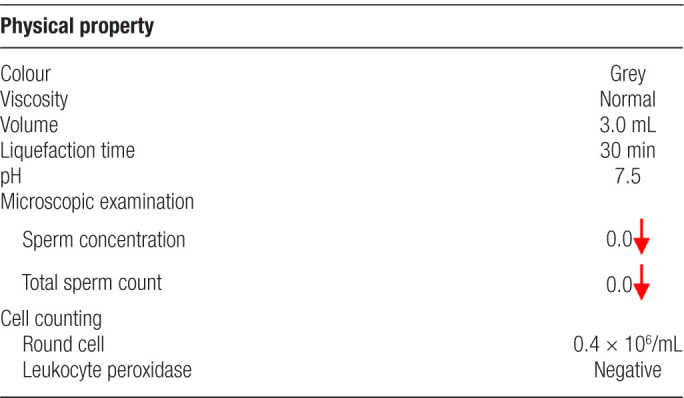
Routine semen analysis.

**Figure 2.**
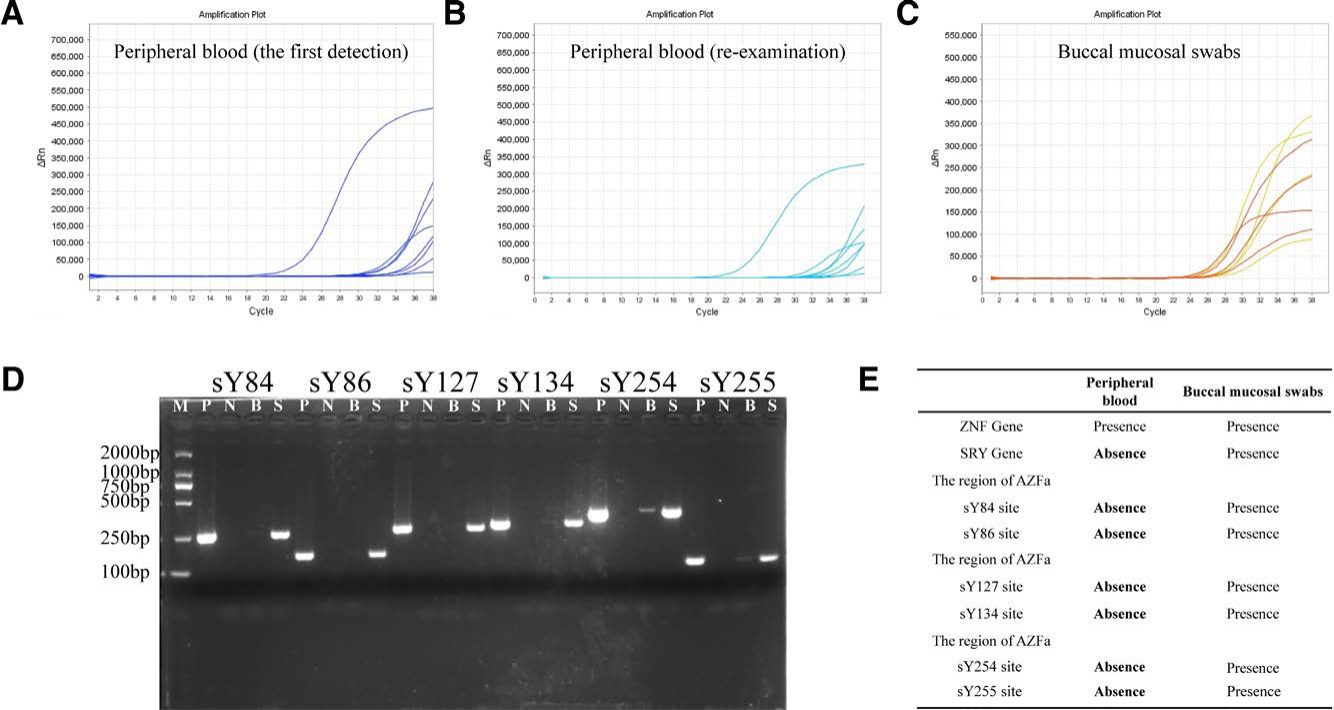
The results of SRY and Y-chromosome microdeletion for the patient’ s (A) in first peripheral blood, (B) in reexamination peripheral blood and (C) in buccal mucosal swabs, respectively. (D) The result of Y chromosome microdeletion in two specimen types by single-pair primer electrophoresis analysis. M: marker; P: positive; N: negative; B: peripheral blood; S: buccal mucosal swabs. (E) Patient’s test report.

The results of SRY and Y-chromosome microdeletion for the patient’ s first peripheral blood test were shown in Figure 2A. As the amplification curves at each point are in the critical range (the cycle threshold value is between 34 and 36), the results cannot be accurately interpreted. To eliminate the abnormal results caused by the operation, we re-extracted, amplified and reexamined the original samples, and the results were the same as before. At this point, we reviewed the patient’s history and only found that the patient had received a BMT from an opposite-sex donor for CML 14 years ago. Since the molecular biological results of Y-chromosome microdeletion suggested the possibility of female chromosome chimerism or that the case itself was a possibility of female inversion, the patient was notified by phone to come to our hospital for reexamination, and 3 mL peripheral blood and buccal mucosa swabs on both sides of the mouth were collected for re-testing. The patient’s peripheral blood test results were the same as the first time (as shown in Fig. 2B), while the buccal mucosal swabs on both sides of the mouth showed a normal male chromosomal phenotype (Fig. 2C). We further verified the Y-chromosome microdeletion of the two specimen types by single pair electrophoresis analysis of specific primers again (Fig. 2D), and the report sheet issued to the patient was shown in Figure 2E.

## 3. Discussion

In this study, we described a male patient who underwent a BMT after being diagnosed with CML 14 years ago, who recently came to our hospital for further treatment after unexpectedly finding no sperm in his semen during a physical examination. Combination with the abnormal semen test indicators, the result of 46,XX karyotype, the completely different Y-chromosome microdeletion results in peripheral blood and somatic cells, it was learned only after detailed medical history that the BMT donor previously received by the patient came from female volunteers of the China Bone Marrow Bank. In other words, the abnormal chromosome karyotype and Y-chromosome microdeletion results shown in the peripheral blood of the patient were derived from the hematopoietic stem cells of the donor, which fully confirmed that the hematopoietic function of the patient has been effectively rebuilt through BMT, so that the patient’s CML has reached the curative level in a long time. However, since the patient’s peripheral blood genetic information at this time showed the chimeric type of donor and recipient, it is not sufficient and accurate to judge the patient’s sex or fertility based on this result. The buccal mucosa swabs on both sides of the mouth mainly detect the somatic cell gene information of the patient, which originated during the embryonic stage and can directly reflect the recipient’ s own chromosome characteristics. This case reminds us that a correct diagnosis of patients depends on the comprehensive collection of present and past medical history, complete physical examination, and careful evaluation of related adjuvant tests. Doctors should never make a one-sided diagnosis based on just one or two test results.

Furthermore, although the structure of the Y chromosome is preliminarily confirmed, it is unknown whether the patient’s fertility is affected, because the critical aspect is the effect of the transplant on the recipient’ s sexual function and reproductive health.^[14]^ There are two reasons: on the one hand, the patient must be pretreated with total body irradiation,^[15]^ chemotherapy and immunosuppression before BMT, which are often part of the transplant process, can have significant impacts on sexual function.^[16–18]^ In this case, the patient was only 8 years old at that time, which was bound to have a great impact on the development of gonads during puberty. On the other hand, medications for chronic conditions or individual differences in the metabolism of therapeutic drugs also determine whether their ultimate fertility is preserved or not. Due to the patient being only 22 years old and having no intention of getting pregnant at present, no further medical treatment was given.

We have reviewed many literature and news reports, and in recent years, with the improvement of treatment methods for blood diseases, there are many cases of sex chromosome abnormalities after allogeneic BMT from an opposite-sex donor. A representative case involved an 18-year-old girl with primary amenorrhea and a 46 XY karyotype, misdiagnosed as Y-chromosome-related disorders of sex development.^[19]^ The patient had normal female reproductive organs and experienced disrupted pubertal development after treatment for acute myeloid leukemia and BMT from her brother. Not surprisingly, many people have even questioned whether the detection of abnormal sex chromosomes means that the patient’ s actual sex has also changed? This is not true, genetic sex is determined when the ovum is fertilized with sperm containing X or Y chromosomes, and the fetus can be identified as male or female by ultrasound when it reaches 12 weeks of development. The presence of the Y chromosome will guide testicular development through the sex-determining region of Y-chromosome (SRY gene), which is located on the short wall. For the male patients in this case or other individuals who have received sex-mismatched BMT, their physiological gender was already determined as early as the moment of sperm-egg fusion, which is not affected by an individual’s disease or treatment history. Instead, the detection of sex chromosomes after sex-mismatched BMT can assist in judging the effectiveness and guide the use of immunosuppressants.

In cases of sex-mismatched BMT, the post-BMT karyotype fully changing to that of the donor’ s origin demonstrates the complexity of gender-related health outcomes in BMT. From another perspective, to more intuitively identify whether healthy hematopoietic stem cells have successfully engrafted in the new environment, doctors increasingly attempt to select donors of the opposite sex for leukemia patients.

## 4. Conclusion

As survival rates for hematopoietic stem cell transplantation improve, there is subsequently an increased need for addressing long-term complications. This case report demystifies some of the misconceptions surrounding the gender of patient post-heterosexual BMT. The findings suggest that while heterosexual BMT can induce some changes in gender-specific traits, these changes are generally limited and do not fundamentally alter the recipient’ s gender.

We hope that through cases like this, clinicians will be alerted that an accurate diagnosis depends on comprehensively collecting present and past medical history, performing a complete physical examination, and carefully evaluating relevant ancillary tests. The diagnosis should never be made blindly based on only one or several test indicators. We also hope that patients who have received heterosexual BMT will not be anxious about this. The presence of opposite-sex chromosomes serves as a useful marker for monitoring engraftment success, but it does not lead to genotypic changes that result in phenotypic abnormalities.

## Acknowledgments

We thank all the participants in this study and all clinicians who were involved in the collection.

## Author contributions

**Data curation:** Li Chang.

**Formal analysis:** Guanglu Che.

**Funding acquisition:** Xiaojuan Liu, Fang Liu.

**Investigation:** Xiaojuan Liu.

**Methodology:** Shuyu Lai.

**Supervision:** Qiuxia Yang, Jiaxin Duan, Fang Liu.

**Writing – original draft:** Jie Teng.

**Writing – review & editing:** Xiaojuan Liu, Li Chang, Guanglu Che, Qiuxia Yang, Shuyu Lai, Jiaxin Duan, Hui Jian, Fang Liu.
